# The Impact of Social Conformity on Adopting Decision of Shared Electric Vehicles: A Choice Experiment Analysis in China

**DOI:** 10.3390/ijerph19041955

**Published:** 2022-02-10

**Authors:** Wenbo Li, Mengzhe Wang, Miao Yu, Xiao Zheng

**Affiliations:** Business School, Jiangsu Normal University, Xuzhou 221116, China; liwenbo@jsnu.edu.cn (W.L.); 2020210067@jsnu.edu.cn (M.W.); 3020180329@jsnu.edu.cn (M.Y.)

**Keywords:** social conformity, shared electric vehicle, choice experiment method, logit model

## Abstract

Social conformity, a psychological phenomenon commonly shared by most individuals, has long been ignored by studies focusing on influencing preferences for shared electric vehicles (SEVs). To fill this gap, this paper divides social conformity into informational conformity and normative conformity, and analyzes their effects on individuals’ choice of SEVs. Respondents were selected randomly in Jiangsu Province, and the data were collected by the choice experiment method. The data were further analyzed by logit models. Results show that social conformity has a significant positive impact on individuals’ choice of SEVs, and informational conformity has a much more profound impact than normative conformity. The driving cost and the convenience of picking up and returning a vehicle also influence consumers’ preferences. In addition, social conformity cannot totally dispel the negative impact of poor experience. Finally, some targeted policy recommendations are proposed.

## 1. Introduction

**Figure 1:**
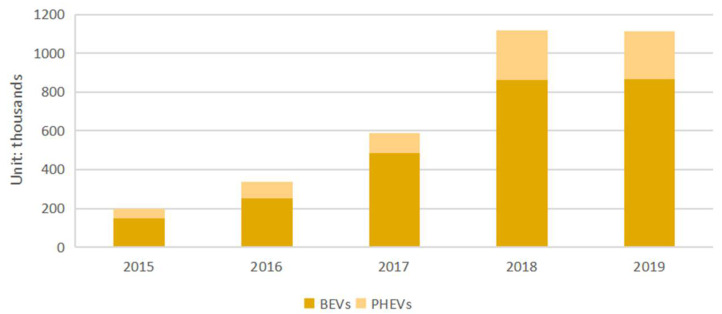
New electric vehicle sales (thousands).

With the rapid development of the global economy, energy consumption has been rising year by year, which has accordingly led to a sharp increase in carbon emissions. At the end of 2018, global carbon emissions had reached 33.243 billion tons, while China’s carbon emissions account for about 27% of global carbon emissions. China’s total carbon emissions from the transportation sector are around 1.1 billion tons, accounting for about 10% of the country’s total carbon emissions [1]. More critically, transportation has a wide variety of important environmental impacts, which globally contribute to fossil fuel dependence, global warming, and environmental degradation [2]. The transport sector produces three major greenhouse gas emissions (CH_4_, CO_2_, and N_2_O) and five air pollution emissions (NH_3_, NO_x_, PM2.5, SO_2_, and volatile organic compounds) [3]. Particulate matter (PM) is considered to be one of the most important air pollutants, causing or contributing to a large number of adverse health effects associated with pollution [4]. As early as 2010, 55% of N_2_O emissions from the transportation sector were generated by catalytic emission control systems for light-duty vehicles [5]. Despite the recent adoption of more stringent vehicle emission regulations in many countries, the transport sector remains a major source of the global air pollution disease burden [6]. This has attracted the attention of many countries, institutions, and scholars to actively seek strategies to increase the diversity of transport systems and reduce vehicle travel [7]. In recent years, facing the challenges of the global energy crisis and environmental pollution, the Chinese government has announced a variety of policy measures, some of which have been implemented, including enhanced monitoring and reporting [8], emphasizing the importance of developing public transportation, and advocating green travel [9]. China has stepped up its efforts to explore environmental protection in the automotive sector and vigorously promote the development of EVs [10]. Driven by the goals of “emission peak” and “carbon neutrality”, China has accomplished a thriving production and sales performance of EVs, with the number of EVs in China reaching 3.384 million by 2019 [11]. China’s EV industry has entered the growth phase from the incubation period and has become an important force in leading the transformation of the global auto industry. Figure 1 shows the number of electric vehicles sold in China from 2015 to 2019 [1].

**Figure 2:**
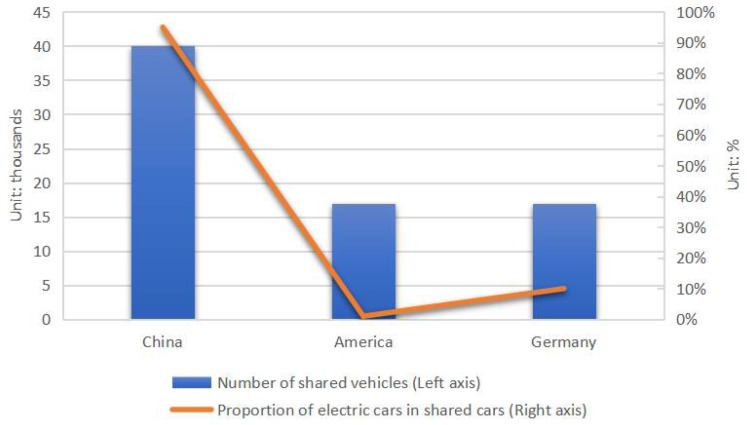
Number of shared vehicles and proportion of electric vehicles in shared vehicles.

However, compared to the current car ownership of over 300 million in China, the popularity of EVs is still far from enough. In order to increase the adoption rate of EVs, reduce the impact of carbon emissions in the transportation sector, and solve a series of problems caused by high car ownership (such as high fuel prices, urban traffic congestion, limited social resources per capita, increasing costs of buying and maintaining cars, urban parking spaces in short supply, and environmental protection problems), the idea of changing the traditional production and sales model of automobiles comes into being. The emergence of the “sharing economy” has led to a more rational use of resources [12]. With the spread of digital technologies and the unprecedented efficiency of coordinating access to resources, new sharing models and practices have emerged in the space of traditional sharing and formal market economies, leading to the emergence of a new class of resource allocation systems that are also called the “digital sharing economy” [13]. According to statistics, each 80-mile range shared autonomous EV can replace 3.7 private vehicles and each 200-mile range shared autonomous EV can replace 5.5 private vehicles, under 240 volt AC charging [14]. As a result, once the use of car sharing as a supplement to public transportation is promoted, it can greatly improve the current traffic situation, relieve the pressure on infrastructure, and improve the efficiency of resource utilization. At the same time, for consumers, car sharing can meet their daily work and life needs while eliminating a series of additional expenses such as repair costs, maintenance costs, and insurance premiums. From Figure 2, we can see the number of shared vehicles and proportion of electric vehicles in shared vehicles in different countries.

Car sharing has been gaining traction in Europe and the United States. In Germany’s capital city, Berlin, car sharing has grown to city-wide popularity. Currently, about 46% of Berliners do not own a private car. Car sharing in Germany and other European and American countries has a decades-long history of development and has become synonymous with easing urban traffic and being efficient and environmentally friendly. However, shared electric vehicles (SEVs) are still in their infancy in China. From 2017 to 2018, Didi, Mobai, and Trip.com Group launched car-sharing services one after another, basically covering first-tier cities such as Beijing, Shanghai, and Guangzhou. However, the final statistics are not optimistic. On the one hand, many people download the car-sharing app, but do not really integrate it with their lives, and often just abandon it after experiencing it. Of course, this is probably because the car-sharing service itself is flawed and needs to be improved to enhance the customer experience. On the other hand, more people are still unable to accept the new car-sharing service from their hearts, mainly because few people or no one around them uses it. Based on this, this paper intends to study the influence of social conformity on expanding the market of car-sharing services. Crutchfield [15] believed that individuals will consciously or unconsciously succumb to the pressure of those around them and thus do what is consistent with the popular position. This phenomenon, known as social conformity, has been studied and discussed since the mid-nineteenth century. Social conformity arises from two main sources, and they are informational conformity and normative conformity. Informational conformity refers to people making choices that are consistent with the choices of others around them when they lack control over the situation and are unsure which is the right choice. Normative conformity refers to people having a desire to be accepted or liked by the group and people wanting to be right. The influence of social conformity on individual behavioral choices is very strong; Asch [16] was surprised to discover through a series of experiments that people sometimes behave in the same way even when they know that others are not doing the right thing, due to group pressure and the cost of being “different”. Therefore, it is important to look at individuals’ acceptance of the emerging electric car sharing from the perspective of social conformity.

Considering that individuals’ preferences for SEVs are influenced by both experience and social conformity, this paper will explore the influence of attributes and attribute levels of SEVs and social conformity on individuals’ acceptance through a choice experiment method, a type of declarative preference method. Based on the results of this paper, policymakers and companies could develop and improve policy strategies and marketing tools to guide the public to change their attitudes and help achieve energy saving and emission reduction goals. It is expected to provide new insights for the promotion of SEVs at the micro level, and provide suggestions to the government as well as SEV service providers.

The remainder of this paper is organized as follows: Section 2 presents the main strands of the related literature. Section 3 contains the survey design, data collection, and attributes and attribute levels used in the experiment. Estimation results are discussed in Section 4. Section 5 concludes the paper.

## 2. Literature Review

Many scholars in China and abroad have studied and observed individuals’ willingness to use EVs. Based on the theory of planned behavior (TPB), Zhao et al. [17] introduced perceived risk and perceived value to construct an integrated model. After an empirical study, subjective norms were found to have the greatest impact on individuals’ willingness. By conducting a choice experiment and random parameter logit model, Wang et al. [18] found that transport policy incentives have the most significant positive effects on EV acceptance, and discounted/free electric charging also makes a great contribution. Ščasný et al. [19] found that EVs are less preferred than conventional cars. However, decreasing the purchase price and operating costs, updating skills that increase the driving range, and decreasing charging time can all serve to strengthen preferences for EVs. Some studies have also analyzed the willingness to use SEVs. For instance, Zhang et al. [20] took an alternative approach to probe solutions of improving the service quality and synergy between SEVs and traditional urban public transportation from the perspective of travel cost and comfort in residents’ travel utility. The ultimate goal is to share EVs to improve the existing traffic situation and enhance the travel utility of residents. Kim [21] examined the effects of the activity-travel context and individual latent attitudes on carsharing decisions under travel time uncertainty and used a hybrid choice modeling framework to estimate the effects of all these factors simultaneously. The findings show that time constraints and lack of spontaneity have significant effects on people’s intention to use a shared car. Abotalebi et al. [22] reviewed the development of EVs through a Canadian survey collecting over 20,000 observations across Canada. The survey captured household preferences and behavioral intentions toward EVs through choice experiments and a comprehensive, integrated series of attitudinal questions.

In summary, most of the current studies focusing on the factors influencing individuals’ preference for SEVs are concerned more about the convenience and cost of use, parking, and other specific details of use and the sense of use experience. Yang et al. [23] identified subsidy policies and pricing strategies of car-sharing operators as key factors affecting the large-scale adoption of car-sharing and proposed a subsidy and pricing model for electric car-sharing based on a two-stage Stackelberg game. Illgen et al. [24] studied the competitiveness of EVs in car-sharing networks and concluded that the pivotal success factors include favorable relationships between market environments (e.g., electricity and fuel prices) and important characteristics of EVs (e.g., price and range). Some scholars have also begun to analyze the possibility of influencing personal preference for SEVs from the perspective of psychology, but the existing studies have ignored social conformity, which is also one of the psychological factors. Studies that discuss the influence on personal willingness to use SEVs from the perspectives of informational and normative conformity are rare. By summarizing the existing articles, it is easy to find that there are many articles in the fields of environment and energy combining social conformity and individual behavior. The successful implementation of the recycled drinking water (RDW) case demonstrates the importance of trust, information, and social norms in changing people’s psychological discomfort with RDW. Based on this case, Leong et al. [25] used a simple choice experiment to further conclude that social norms, i.e., consistency itself, are sufficient to change people’s attitudes and behaviors toward RDW. Li et al. [26] proposed a game model related to individuals’ green behaviors, and divided the relationship between individuals into two categories of strong and weak links. They also set some parameters associated to individuals’ green behaviors (IGBs), such as temptation benefit, stubbornness index, and choice preference. Finally, it is concluded that when people show higher connectivity or a lower activity rate, they are more inclined to adopt green behaviors. That is, it is suggested that the government or companies should guide others by directing some influential people to take green actions, or further strengthen the publicity to enhance public awareness of environmental protection. In conclusion, social conformity has an extremely strong influence on individual behavioral awareness, and the influence of conformity on individuals’ preferences for SEVs cannot be ignored.

The choice experiment method, as a type of stated preference method, is suitable for solving the problem of how to achieve the optimal level of welfare for society from a society-wide perspective. A number of previous studies have used the choice experiment method to assess consumers’ demand preferences. The choice experiment method conducted by Cherchi [27] contained several EV attributes (two policy attributes and two dummy attributes) to analyze the influence degree of individuals on EV preferences, but the coverage of vehicle-specific attribute is incomplete. Helveston et al. [28] modeled consumer preferences for conventional vehicles, hybrid EVs, plug-in hybrid EVs, and battery EVs in China and the U.S. by using data from choice-based conjoint surveys carried out in 2012–2013. Beck and Rose [29] creatively combined the best–worst scaling and choice experiment method. They concluded that the energy crisis, climate change, and air quality problems all affect people’s attitudes toward mileage, thus changing people’s travel behavior. In a survey of Dutch private vehicle owners, Hoen et al. [30] found that they have negative attitudes toward alternative fuel vehicles, especially for electric and fuel-cell vehicles due to their limited driving distances and longer refueling times. Public preferences for alternative fuel vehicles increased substantially with improvements in driving distance, refueling time, and fuel availability. Parsons et al. [31] examined potential consumer demand for vehicle-to-grid EVs using data from a national stated preference survey. In a choice experiment, 3029 respondents compared their preferred gasoline vehicle with two vehicle-to-grid EVs. The results suggest that the vehicle-to-grid concept is likely to help achieve the diffusion of EVs. Jensen et al. [32] studied the effect of real-life experience with EVs on individual preferences and attitudes over a relatively long period of time. The stated choice method is used to stimulate public preferences for the situation where EVs and related charging facilities are not yet fully integrated into the market.

Given the above, with the goal of making up for the shortcomings of previous studies, this paper combines SEV attributes such as the convenience of picking up and returning vehicles and social conformity, which is divided into informational conformity and normative conformity, to study individuals’ preferences for SEVs. At the same time, few studies have used this method to analyze the choice behavior of Chinese consumers in relation to SEVs, as most of the existing studies using choice experiments have been conducted on consumers in developed countries such as Europe, the United States, Korea, and Japan. This study will also fill this gap.

## 3. Methods

The choice experiment is mainly based on random utility theory and the factor value theory proposed by Lancaster. This theory assumes that any good can be described by a set of attributes, and different levels of these attributes. An important feature of this approach is the ability to examine individual choice preferences in different hypothetical contexts. Given that most people are unfamiliar with SEVs, which are emerging technology products, it makes sense to use the choice experiment approach for the research discussion. According to this paper, respondents are told to choose between the two SEVs that are the combination of different attributes and attribute levels. Respondents should combine the attributes and attribute levels of each SEV, weigh them with their own needs, and carefully compare them before making a choice. The following is a discussion of each part of this paper’s choice experiment and the detailed elaboration of each part.

### 3.1. Setting Up the Context

In order to explore the effect of social conformity on individual choice preferences, previous researchers such as Cherchi [27] included the measured attribute of social conformity in choice experiments, along with other attributes describing vehicle characteristics for comparison. The present study built on that approach while including three separate scenarios (namely, positive comments, negative comments, and no comments) before respondents made their choices. This new approach was expected to further draw specific effects of informational conformity on individuals’ preference for SEVs. To help respondents better understand the three scenarios, each scenario was described in detail prior to the experiment, with the following wording.

#### 3.1.1. Positive Comments

You are in a social environment where consumers have relatively positive feelings about the use of SEVs. The daily TV news, WeChat push, Tiktok live-streaming, and Weibo comments are basically positive information as long as they are about SEVs, and you have seen the following reports from reporters randomly interviewing consumers about their experiences with SEVs (Table 1). Now, assuming you are in such a context, please complete the following questions.

#### 3.1.2. Negative Comments

You are in a social environment where consumers have relatively negative feelings about the use of SEVs. The daily TV news, WeChat push, Tiktok live-streaming, and Weibo comments are basically negative information as long as they are about SEVs, and you have seen the following reports from reporters randomly interviewing consumers about their experiences with SEVs (Table 2). Now, assuming you are in such a context, please complete the following questions.

### 3.2. Attributes and Attribute Levels

The setting of attributes and their levels is an important part of the choice experiment, which follows three main criteria. First, the attribute sets the need to reflect on the main characteristics of the target product. Due to the shared nature of SEVs, there are some specific concerns. That is, users need to reserve a car in advance based on the distance of the surrounding EV pickup and return points, consider the number of available shared vehicles, and estimate the driving cost based on the distance traveled and the time cost. Therefore, in previous studies, the driving cost, range, and convenience have been taken into account. A choice experiment related to shared vehicles was designed by Kim et al. [21], and they concluded that time constraints, lack of spontaneity, and large changes in travel time all have significant negative effects on the choice preferences of shared vehicle users. Therefore, after taking into account the characteristics of SEVs, this paper set three basic attributes that describe the main characteristics of vehicles: driving cost, range, and convenience. Two additional attributes that have direct impacts on users’ service experience were added and they were vehicle age, and exterior and interior neatness. The different levels of driving cost were set after reviewing the relevant literature and synthesizing the price levels set by different SEV service providers in several cities. Second, the number of attributes sets should be limited, commonly to no more than 10 attributes [33], otherwise it will lead to an overly complicated experimental task, which is not conducive to the implementation of the experimental task. Therefore, only five attributes were finally selected for this experiment. Third, the attributes set in the experiment should be combined with the research purpose. Because this paper is dedicated to studying the influence of social conformity on individuals’ preferences for SEVs, we added the attribute describing normative conformity, i.e., “the proportion of users in your city”, to compare with other attributes describing the characteristics of SEVs. This also echoed the positive and negative comments reflecting informational conformity in the above scenario, which further perfected the study of the influence of social conformity on personal choice preference. Please see Table 3 for details of the attributes and attribute levels.

### 3.3. Experimental Design

The purpose of the choice experiment was to investigate individuals’ preferences for SEVs under the influence of social conformity. Based on this, the questionnaire was designed in three parts: the first part was a statement of the three choice contexts in the scenario, and the respondents were informed that all subsequent choices were made in the context. The second part was a detailed explanation of the concept of “sharing” and an introduction to the use of SEVs, to ensure that respondents had a comprehensive and clear understanding of SEVs before the experiment began. The third part was the content of the experiment. If the attributes and levels set in Table 1 were combined directly, tens of thousands of combinations could be obtained. It was unrealistic to present them all in the questionnaire. Therefore, it was necessary to reduce the dimension of attribute combinations, and filter out the representative product combinations to present to the respondents. We used the orthogonal experiment design function of SPSS to obtain sixteen sets of representative experimental tasks (see Table 4 for an example), each consisting of two SEVs with completely different attributes, and respondents were required to choose one of them or neither of them.

### 3.4. Data Collection

First, the questionnaire was designed according to the choice experiment method, and interviews were conducted with relevant experts and consumers of SEVs to adjust the questions to ensure that the questionnaire was easy to understand and had no logical errors, as well as to ensure that the respondents understood the items correctly. Second, to ensure and verify that the questionnaire had good credibility, a pre-survey was conducted in January 2021, and 30 questionnaires were distributed and collected. The questionnaires were further processed based on the results of the pre-survey to form the final questionnaire. Subsequently, the questionnaires were distributed mainly in Nanjing, Xuzhou, Wuxi, and Suzhou. Jiangsu Province was chosen because by the end of 2020, the province had promoted 274,000 EVs, and the proportion of new or updated EVs in the public transport sector had exceeded 90%. Over 125,000 charging infrastructure supervision and operation platforms and charging piles of various types have been built, and 198 highway service areas and 1073 townships in the province have achieved full coverage of charging piles. Therefore, it is more meaningful and feasible to study the issue of residents’ preference for SEVs in Jiangsu Province. Using a sample size calculation, which was proposed by Johnson and Orme based on the number of attributes and attribute levels, the minimum sample size advised for this study was at least 125 [34]. The equation depending on the number of choice tasks (t), the number of alternatives (a), and the number of analysis cells (c), is as follows:

N > 500c/(t × a)

A target of 200 was identified to allow for incomplete or inconsistent data. A total of 454 questionnaires were distributed and collected, of which 135 invalid questionnaires (30 offline and 105 online) were excluded, and 319 valid questionnaires (30 offline and 289 online) were retained. The effective recovery rate was 70.26%, which met the recovery requirement. The sample statistical characteristics of this survey are shown in Table 5.

It can be seen that 69.9% of the respondents are male and 30.1% are female; the number of respondents aged 18–25 is higher at 35.4%; the annual household income of the respondents spans a wide range, with the largest number of respondents having ¥100–200,000, accounting for 37% of the total; the proportion of households with three members is 17.6%; 29.4% of the respondents have only one car at home; and the vast majority of respondents do not drive more than 50 km per week, accounting for 71.8% of the respondents.

## 4. Results and Discussion

**Figure 3:**
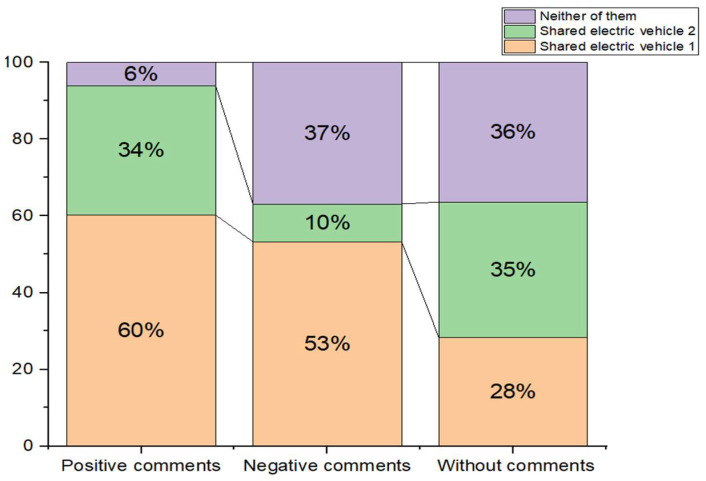
Users’ willingness to choose SEVs under different comment scenarios.

In the three scenarios designed for this experiment, we found that 93.88% of users would choose SEVs in the positive comment scenario (60.21% of them chose SEV 1 and 33.67% of them chose SEV 2); 63.12% of people chose SEVs in the negative comment scenario (53.28% of them chose SEV 1 and 9.84% of them chose SEV 2); and 63.63% of people chose SEVs in the scenario without any oriented information evaluation (28.28% of them chose SEV 1 and 35.35% of them chose SEV 2). It can be found that in the same question setting scenario, the negative comment has a greater impact on the willingness to choose SEVs than the positive comment, so it is difficult for the positive comment to completely offset the impact of the negative comment caused by the short range and the small distribution of outlets. The following is a percentage stack histogram of user choice for each scenario (Figure 3).

In this study, Stata econometric software was used to fit the regression to the obtained data. In order to ensure the validity of the model, two models were constructed for comparison, and the optimal model was finally selected. Among them, model 1 is the base model, which only considers the attributes involved in the choice experiment and their attribute levels without considering the influence of demographic characteristics. Model 2 further includes gender, age, household income, household size, number of electric cars owned by households, number of ordinary cars owned by households, and environmental awareness to measure the influence of demographic characteristics on respondents’ choice of shared electric cars. Table 6 shows the results of the estimation of the two different logit models. It can be seen that Model II improves the overall fit of the model after introducing demographic variables. From the regression results, the value of the likelihood function of Model II (−1002.684) is significantly larger than that of Model I (−1255.441). Thus, Model II is more suitable for analyzing the obtained data.

In particular, the coefficients of convenient pick-up and return, inconvenient pick-up and convenient return, and convenient pickup and inconvenient return were estimated as compared to inconvenient pick-up and return. The coefficients of clean exterior and interior, clean exterior and dirty interior, and dirty exterior and clean interior were estimated as compared to dirty exterior and interior. However, since the *p*-values for clean exterior and dirty interior, dirty exterior and clean interior are not significant, they are not included in the discussion in the following.

In terms of the sign of the coefficients, range, convenient pick-up and return, convenient pick-up and inconvenient return, inconvenient pick-up and convenient return, clean exterior and interior, the number of users in your city, and positive comments will increase consumers’ utility, while driving cost, vehicle age, and negative comments will decrease consumers’ utility. In terms of the absolute value of the coefficients, a higher absolute value indicates a higher respondent preference for the attribute. The results show that the respondents’ preferences for each attribute of SEVs vary significantly; in descending order: driving cost, negative comments, convenient pick-up and return, positive comments, convenient pick-up and inconvenient return, inconvenient pick-up and convenient return, clean exterior and interior, vehicle age, the number of users in your city, and range. The results are in line with the recent research by Wang [35], the likelihood of using shared automatic cars depends mainly on operating costs and hourly rates. Mounce et al. [36] also found that vehicles being accessible when and where users need them matters, which is the key to the success of shared vehicle systems. From the model estimation results, it is clear that positive and negative comments are preferred by respondents over the proportion of users in their city, i.e., they are more likely to influence the choices made by respondents. This shows that informational conformity has a greater influence on individuals’ preference for SEVs than normative conformity. Particularly, the influence of negative comments should not be underestimated, suggesting that when society is full of negative comments about SEVs, a large number of potential users will be resistant to SEVs and even give up trying them. This is where much of the current literature examining shared electric vehicles has been ignored. Among the demographic characteristics included in the model, all three variables have significant negative effects in addition to the insignificant results of the gender and income, which indicate that SEVs are more likely to be favored by consumers who are younger, have a smaller household size, and have fewer cars in the household. Young people were also found to be more open to shared travel options in the study by Tian [37].

These findings not only fill in the gaps in existing research on the social conformity of SEVs, but also help policy makers as well as designers to comprehensively understand the needs of different groups, in order to make policy adjustments and changes in design and marketing strategies. For example, marketing strategies may initially be more skewed toward younger groups. Another example is that SEV service companies should pay enough attention to negative feedback from the community about the rental service. However, current research is based on preference data and lacks empirical research on the behavior of people who actually choose SEV services, as constantly updated technological innovations and marketing efforts may make people more willing to accept this emerging service, so this is part of the direction of our future work.

## 5. Conclusions

In this paper, we applied a choice experiment combined with a random parameter logit model to analyze the effect of social conformity on individuals’ preference for SEVs. The main findings are as follows. First, both informational and normative conformity have positive impacts on individuals’ preference for SEVs, and informational conformity has a more significant effect. Second, consumers are concerned more about the cost and convenience of SEVs, and less concerned about the vehicle age, interior and exterior cleanliness, and range. Then, even if the whole social environment is positive about SEVs, it is difficult to compensate for the negative impact caused by high cost of use, inconvenience of pick-up and return, and so on. Finally, individuals who are young, have a small family size, and own a small number of cars at home are more likely to be a potential group for using SEVs.

Based on the above findings, the following recommendations are made. First, the government and enterprises should pay attention to the role of social conformity in the process of promoting SEVs, especially the influence of informational conformity. This can be used as a basis to formulate policy strategies and marketing strategies, such as inviting credible people to make public service announcements to guide people to consciously change their travel patterns, and promote this new low-carbon travel mode on major media platforms, such as Tiktok, Sina-Weibo, and Bilibili, which have high traffic and exposure rates. This way, more people will know about this new product in a short period of time, which will trigger people’s desire to learn more about and use shared electric cars. At the same time, companies can also offer various discounts to promote SEVs, with a particular focus on the “recommend it to others” marketing model, so that the peer effect and the social conformity effect can win a larger audience. However, in the process of promotion, we should focus on the impact of negative information and respond positively to all kinds of negative information, rather than just ignoring it and avoiding it. Second, efforts should be made to improve the service experience of SEVs. Discount activities can be appropriately conducted in the early stage of business to reduce consumers’ cost of use and motivate them to choose SEVs for daily travel. In addition, the setting of SEV outlets should be more reasonable. The outlets that are too close together will result in wasted resources, and those that are too far away will lead to consumer dissatisfaction. The contradictory relationship between cost and service experience should also be reconciled. Thirdly, we should seize potential customers to perform key promotion and publicity. The promotion and publicity can be focused on young people between 18 and 30 years old, unmarried people without a car, or those who travel a short distance. For instance, advertising can be carried out in places with more young people such as colleges and universities, and SEV outlets can also be set up there.

## Figures and Tables

**Table 1:** Interview Q&A (positive).

Question	Answers
What do you think of the range of shared electric cars?	Consumers: It’s very good and perfectly suited for my travel needs.
Do you think the distribution of car-sharing outlets can meet your daily travel needs?	Consumers: It’s very good and totally satisfying.
Do you think the price of using shared electric cars is reasonable?	Consumers: Yes, I think it’s reasonable.

**Table 2:** Interview Q&A (negative).

Question	Answers
What do you think of the range of shared electric cars?	Not sure, sometimes it cannot satisfy my travel needs.
Do you think the distribution of car-sharing outlets can meet your daily travel needs?	Not sure, sometimes it cannot satisfy my travel needs.
Do you think the price of using shared electric cars is reasonable?	No. I think it’s unreasonable.

**Table 3:** Attributes and attribute level design of SEVs.

Attributes	Attribute Levels
Driving cost	¥0.25/km; ¥0.5/km; ¥0.75/km; ¥1.0/km.
Range	50 km; 75 km; 100 km; 150 km.
Convenience	Convenient pick-up and return;
	Inconvenient pick-up and return;
	Convenient pick-up and inconvenient return;
	Inconvenient pick-up and convenient return.
Vehicle age	0 year; 1 year; 3 years; 5 years.
Exterior and interior neatness	Clean exterior and interior;
	Dirty exterior and interior;
	Clean exterior and dirty interior;
	Dirty exterior and clean interior.
The proportion of users in your city	More than 10%;
	More than 30%;
	More than 50%;
	More than 70%.

**Table 4:** An example of experimental tasks.

Attributes	SEV 1	SEV 2
Driving cost	¥0.25/km	¥1.0/km
Range	100 km	150 km
Convenience	Inconvenient pick-up and convenient return	Convenient pick-up and return
Vehicle age	5 years	3 years
Exterior and interior neatness	Clean exterior and interior	Clean exterior and dirty interior
The number of users in your city	More than 70%	More than 30%
Please choose one vehicle and √ in □	□	□

**Table 5:** Statistical characteristics of samples.

Demographics		Total (N = 319)	
		Frequency	Percentage
Gender	Male	223	69.9
	Female	96	30.1
Whether you have used shared electric cars	Yes	247	77.4
	No	72	22.6
Age (years)	18–25	113	35.4
	26–30	98	30.7
	31–35	47	14.7
	36–45	38	11.9
	46–55	17	5.3
	56 and over	6	1.9
Family annual income	Less than ¥100,000	89	27.9
	¥100,000–¥200,000	118	37.0
	¥200,000–¥300,000	65	20.4
	¥300,000–¥400,000	38	11.9
	More than ¥400,000	9	2.8
Family population	1 person	93	29.2
	2 people	115	36.0
	3 people	56	17.6
	4 people	33	10.3
	5 or more than 5	24	7.5
Number of ordinary cars owned by households	0	166	52.0
	1	94	29.4
	2	49	15.4
	3 and over	10	3.1
Number of electric cars owned by households	0	253	79.3
	1	66	20.7
	2	0	0
	3 and over	0	0
Miles driven per week (km)	0–50	229	71.8
	50–100	63	19.7
	150–200	22	6.8
	200 and over	5	1.5

**Table 6:** Estimation results of the simulated choice intention model.

Variables	Model 1		Model 2	
	Estimated Coefficient	*p*	Estimated Coefficient	*p*
Driving cost	−1.791	0.000	−1.889	0.000
Range	0.004	0.000	0.003	0.003
Convenient pick-up and return	1.452	0.000	1.541	0.000
Convenient pick-up and inconvenient return	0.775	0.000	0.799	0.000
Inconvenient pick-up and convenient return	0.751	0.000	0.723	0.000
Vehicle age	−0.039	0.016	−0.059	0.002
Clean exterior and interior	0.741	0.000	0.626	0.000
Clean exterior and dirty interior	0.075	0.568	0.064	0.625
Dirty exterior and clean interior	−0.269	0.056	−0.199	0.067
The number of users in your city	0.009	0.000	0.005	0.046
Positive comments	0.179	0.011	0.941	0.009
Negative comments	−1.097	0.012	−1.881	0.029
Gender	/	/	0.002	0.999
Age	/	/	−0.107	0.011
Income	/	/	0.005	0.850
Family population	/	/	−1.053	0.006
Number of cars owned by households	/	/	−0.9819	0.030
Log likelihood	−1255.441		−1002.684	
